# The effects of arginine supplementation through different ratios of arginine:lysine on performance, skin quality and creatine levels of broiler chickens fed diets reduced in protein content

**DOI:** 10.1016/j.psj.2022.102148

**Published:** 2022-08-27

**Authors:** Carlos H. Oliveira, Kelly M.M. Dias, Romário D. Bernardes, Thiago F. Diana, Ramalho J.B. Rodrigueiro, Arele A. Calderano, Luiz F.T. Albino

**Affiliations:** ⁎ Department of Animal Science, Universidade Federal de Viçosa, 36570-900, Viçosa - MG, Brazil; † CJ Corporation, Av. Engenheiro Luís Carlos Berrini, 04571-010, São Paulo – SP, Brazil

**Keywords:** essential amino acid, reduced crude protein content, broiler, skin quality, creatine level

## Abstract

Two trials were carried out to assess the effects of arginine supplementation through ratios of digestible arginine:lysine on growth performance, skin quality and creatine levels in muscle and serum of broiler chickens fed diets reduced in protein content. A total of 1,540 Cobb500 male chickens were distributed into 7 treatments, with 10 replicates with 22 birds each. The experimental diets were based on corn and soybean meal, and a control diet was formulated to satisfy broiler nutritional requirements. A basal diet with reduced protein content was formulated to meet broiler nutritional requirements, except for SID Arg levels. The experimental diets were obtained by adding L-arginine to basal diets, meeting 6 different SID Arg:Lys ratios (94, 100, 106, 112, 118, and 124%). Body weight, body weight gain, average daily feed intake, and feed conversion ratio were evaluated from 01 to 21 d old (trial 1) and from 22 to 44 d old (trial 2). At 21 and 44 d, in trials 1 and 2, respectively, birds were slaughtered to assess skin thickness (**ST**), skin strength (**SS**), creatine level in muscle (**CRM**) and serum (**CRS**). Data were subjected to ANOVA, and treatments were compared to the control group by Dunnett's test (*P* ≤ 0.05). Regression analyses were performed to model the variables assessed and the ratios of SID Arg:Lys. The SID Arg:Lys ratios did not affect ADFI of broilers in both trials (*P* > 0.05), whereas it linearly increased the BW, BWG, and ST, in both trials (*P* < 0.001). The FCR of broilers linearly decreased, in trial 1 (*P* = 0.038) and trial 2 (*P* < 0.001). The CRM of birds had a linear effect (*P* < 0.001) in trial 1, and a quadratic effect (*P* = 0.001) in trial 2. The CRS and SS of broilers linearly increased, in trial 2 (*P* < 0.001). In conclusion, increasing SID Arg:Lys ratios in diets reduced CP enhanced growth performance, skin quality and CR levels in muscle and serum of broiler chickens from 01 to 21 and 22 to 44 d old.

## INTRODUCTION

For many years, diets have been formulated to meet broiler requirements according to crude protein (**CP**) content, which may result in some amino acid (**AA**) levels above the recommended levels. Consequently, there is considerable interest in the successful development of reduced CP diets associated with feeding costs and decreased nitrogen excretion and environmental pollution (Maia et al., 2021). The inclusion of unbound AAs (crystalline or synthetic) enables a substantial reduction in the protein levels, in which low-protein diets plus unbound AAs are formulated to meet the requirements for all AAs and are expressed as a percentage of lysine, the reference AA (Emment and Baker, 1997). However, tangible reductions in dietary CP can compromise broiler performance even if unbound AAs are supplemented to meet bird requirements, and the ratio of AAs to Lys is not permanent, as it is influenced by various factors such as amino acid antagonism (Chrystal et al., 2020).

Conventionally, AA requirements for broiler chickens have been focused on its primarily functions as a structure in the protein chain. However, recent studies have shown that some AAs play important roles in multiple signaling pathways, thereby regulating gene expression, intracellular protein turnover, nutrient metabolism, immunology and oxidative defense (Wu, 2010; Yu et al., 2022). Among the functional AAs, Arg is one of the most versatile in animal physiology because it is a precursor of several molecules, such as creatine, ornithine, nitric oxide, citrulline, proline, and polyamines (Fernandes and Murakami, 2010; Birmani et al., 2019).

Creatine (**CR**), as a precursor of phosphocreatine (**PCR**), has a direct role in protein accretion by improving the availability of adenosine triphosphate (**ATP**) for myosin (Portocarero and Braun, 2021). The CR:PCR system acts to transfer high-energy phosphate groups from the mitochondrial matrix to the sites of ATP utilization in the cytosol, acting as a reservoir of phosphate that can restore ATP from adenosine diphosphate (**ADP**) (Bender, 2012). Regeneration of ATP is particularly important in fast-growing species such as broiler chickens (Portocarero and Braun, 2021). The biosynthesis of CR is thoroughly understood and requires 3 AAs, Arg, Gly and Met, and 2 enzymes, L-arginine-glycine amidinotransferase (**AGAT**) and guanidinoacetate methyltransferase. Literature regarding broiler chicken responses to dietary Arg levels reported that CR levels in muscle are directly affected by this AA. Thus, the CR level in muscle can be used as a criterion to assess the Arg requirement of birds (Chamruspollert et al., 2002).

Fragile skins can downgrade chicken carcass. Therefore, increases in skin strength are an important economic factor in poultry processing (Kafri et al., 1985). Christensen et al. (1995) reported an improvement in skin strength of female broilers as synthetic L-proline was incorporated into diets. Pro, Gly, and hydroxyproline (**Hyp**) are the main components of collagen type I, a protein that confers strength and flexibility to skin, bones, and tendons, in addition to contributing to wound healing of those tissues (Aykın-Dinçer et al., 2017). A study carried out by Austic (1973) indicated that chicks fed a Pro-free diet converted approximately 7% of the dietary Arg to Pro. Therefore, Arg may contribute to improving the skin quality of broilers.

Arg plays an important role in broiler growth performance, participating in protein synthesis either directly as a monomer in the protein chain or indirectly as taking part in multiple metabolic pathways (Yao et al., 2008; Fernandes and Murakami, 2010). There is evidence that Arg is a powerful secretagogue that stimulates the release of growth hormone (**GH**) and insulin-like growth factor 1 (**IGF-1**) in the bloodstream (Silva et al., 2012). Furthermore, Arg acts as a trigger of target of rapamycin (**TOR**) signaling pathway activation, a complex that controls many aspects of cellular physiology, increasing protein synthesis and decreasing protein degradation (Dann and Thomas, 2006; Yao et al., 2008).

Birds do not synthetize Arg efficiently due to the absence of a functional urea cycle, as the excretion of nitrogenous compounds by the uric acid pathway is not Arg-dependent (Bender, 2012). Therefore, Arg is an essential AA and must be supplied by nutritional programs to meet chicken physiological requirements. However, an excessive dietary Arg level could result in a negative effect on broiler physiology due to the antagonism between Arg and Lys, which may reduce food consumption, weight gain and Arg utilization in animal metabolism (Fernandes and Murakami, 2010). The mechanism behind the lysine-arginine antagonism has not been cleanly defined but it does appear to be linked to the reduced capacity for the renal tubes to reabsorb arginine, and reduced arginase activity (Maynard and Kidd, 2022).

Owing to the struggle to determine the Arg:Lys ratio, especially in reduced CP diets due to Arg versatility in bird metabolism and its antagonism with Lys, we carried out the current research to assess the effects of arginine supplementation through different ratios of standardized ileal digestible (**SID**) Arg:Lys on the performance, CR levels in muscle and serum, and skin quality of broilers fed diets formulated with a reduction in CP content.

## MATERIALS AND METHODS

### Ethics Committee

All procedures adopted in this research were previously approved by the Ethics Committee in the Use of Farming Animals at the Federal University of Viçosa (CEUAP-UFV) under protocol 01/2021, in accordance with the norms of the National Council for Experimentation Animal Control (CONCEA, 2008).

### Common Procedures and Experimental Design

The current research was divided into two dose–response trials according to the growth phases of the animals: trial 1 (1–21 d of age) and trial 2 (22–44 d of age). The trials were performed at the Research & Extension Sector for Poultry Production and Nutrition from the Animal Science Department–Center for Agricultural Sciences, Federal University of Viçosa, State of Minas Gerais, Brazil.

In both trials, Cobb500 male broiler chicks (Cobb-Vantress, Inc., East Siloam Springs, AR) were obtained from a commercial hatchery at 1 day old, where all chicks received vaccinations for Marek's disease, Newcastle disease, and infectious bronchitis. Broiler chicks were reared into floor pens equipped with one tubular feeder of 20 kg, 5 in-line nipple drinkers, and a litter system of fresh wood shavings. Experimental diets and water were provided ad libitum throughout the experimental period. Temperature and light programs were set according to genetic guidelines.

A total of 1,540 chickens with initial body weights of 37.25 ± 2.4 g in trial 1 and 958.62 ± 61.8 g in trial 2 were distributed in a completely randomized design in 7 treatments, with 10 replicates with 22 birds each. In trial 2, prior to achieving 22 d, broiler chicks were reared in solid wall houses, equipped with cross ventilation type and stocking density of 38 kg/m^2^, according to conditions established by Cobb500 guideline recommendations and fed with diets formulated according to Rostagno et al. (2017).

### Experimental Diets

The experimental diets were based on corn and soybean meal. A control diet was formulated to cover all nutrient recommendations according to Rostagno et al. (2017), meeting the ratios of SID Arg:Lys of 107% and CP of 23.11% in trial 1 and 20.77% in trial 2 (Tables 1 and 2).

**Table 1:** Control and reduced CP diets formulations in trial 1 from 01 to 21 days old.

Ingredient (%)	Control	Diet reduced CP					
		SID Arg:Lys ratio					
		94	100	106	112	118	124
Corn	58.13	61.91	61.91	61.91	61.91	61.91	61.91
Soybean meal	24.13	19.30	19.30	19.30	19.30	19.30	19.30
Corn gluten meal	6.00	6.00	6.00	6.00	6.00	6.00	6.00
Fish meal	3.00	3.00	3.00	3.00	3.00	3.00	3.00
Plasma	2.00	2.00	2.00	2.00	2.00	2.00	2.00
Dicalcium phosphate	1.50	1.53	1.53	1.53	1.53	1.53	1.53
Limestone	0.88	0.90	0.90	0.90	0.90	0.90	0.90
Salt	0.13	0.13	0.13	0.13	0.13	0.13	0.13
Soybean oil	2.76	1.90	1.90	1.90	1.90	1.90	1.90
L-Glutamic acid, 99%	0.00	0.85	0.85	0.85	0.85	0.85	0.85
Choline chloride, 60%	0.10	0.10	0.10	0.10	0.10	0.10	0.10
Starch	0.00	0.40	0.33	0.26	0.19	0.12	0.05
L-Lysine HCl	0.35	0.36	0.36	0.36	0.36	0.36	0.36
L-Methionine	0.24	0.29	0.29	0.29	0.29	0.29	0.29
Potassium carbonate	0.00	0.25	0.25	0.25	0.25	0.25	0.25
Sodium bicarbonate	0.25	0.25	0.25	0.25	0.25	0.25	0.25
L-Glycine, 98,5%	0.02	0.18	0.18	0.18	0.18	0.18	0.18
L-Threonine, 98,5%	0.04	0.11	0.11	0.11	0.11	0.11	0.11
L-Isoleucine, 98,5%	0.01	0.09	0.09	0.09	0.09	0.09	0.09
L-Valine, 96,5%	0.00	0.09	0.09	0.09	0.09	0.09	0.09
L-Tryptophan, 98%	0.00	0.03	0.03	0.03	0.03	0.03	0.03
L-Arginine, 98,5%	0.13	0.00	0.07	0.14	0.21	0.28	0.35
Mineral Premix^1^	0.13	0.13	0.13	0.13	0.13	0.13	0.13
Vitamin Premix^2^	0.15	0.15	0.15	0.15	0.15	0.15	0.15
Salinomycin, 12%	0.06	0.06	0.06	0.06	0.06	0.06	0.06
BHT^3^	0.01	0.01	0.01	0.01	0.01	0.01	0.01
Crude Protein, %	23.11	21.38	21.38	21.38	21.38	21.38	21.38
Metabolizable energy, kcal/kg	3,150	3,150	3,150	3,150	3,150	3,150	3,150
Calcium, %	0.970	0.970	0.970	0.970	0.970	0.970	0.970
Phosphorus available, %	0.460	0.460	0.460	0.460	0.460	0.460	0.460
Sodium, %	0.218	0.218	0.218	0.218	0.218	0.218	0.218
Potassium, %	0.923	0.923	0.923	0.923	0.923	0.923	0.923
SID Arginine, %	1.344	1.081	1.150	1.219	1.288	1.357	1.426
SID Lysine, %	1.256	1.150	1.150	1.150	1.150	1.150	1.150
SID Methionine, %	0.600	0.600	0.600	0.600	0.600	0.600	0.600
SID Met. + Cys., %	0.929	0.929	0.929	0.929	0.929	0.929	0.929
SID Threonine, %	0.829	0.829	0.829	0.829	0.829	0.829	0.829
SID Tryptophan	0.226	0.226	0.226	0.226	0.226	0.226	0.226
SID Histidine, %	0.521	0.521	0.521	0.521	0.521	0.521	0.521
SID Leucine, %	2.133	2.133	2.133	2.133	2.133	2.133	2.133
SID Isoleucine, %	0.842	0.842	0.842	0.842	0.842	0.842	0.842
SID Valine, %	0.967	0.967	0.967	0.967	0.967	0.967	0.967

1 Trace mineral premix provided per kg of diet: Mn, 58.36 g; Fe, 41.68 g; Zn, 54.21 g; Cu, 8.31 g; I, 0.84 g; Se, 0.25 g.

2 Vitamin premix provided per kg of diet: vitamin A, 9,638,000 IU; vitamin D3, 2,410,000 IU; vitamin E, 36,100 IU; vitamin B1, 2.60 g; vitamin B2, 6.45 g; vitamin B6, 3.61 g; vitamin B12, 15.9 mg; vitamin K3, 1.94 g; pantothenic acid, 12.95 g; nicotinic acid, 39.20 g; folic acid, 0.90 g; biotin, 89.80 mg.

3 Antioxidant Butylhydroxytoluene.

**Table 2:** Control and reduced CP diets formulations in trial 2 from 22 to 44 days old.

Ingredient (%)	Control	Diet reduced CP					
		SID Arg:Lys ratio					
		94	100	106	112	118	124
Corn	65.34	68.77	68.77	68.77	68.77	68.77	68.77
Soybean meal	19.11	14.63	14.63	14.63	14.63	14.63	14.63
Corn gluten meal	5.00	5.00	5.00	5.00	5.00	5.00	5.00
Fish meal	3.00	3.00	3.00	3.00	3.00	3.00	3.00
Plasma	2.00	2.00	2.00	2.00	2.00	2.00	2.00
Dicalcium phosphate	1.09	1.13	1.13	1.13	1.13	1.13	1.13
Limestone	0.63	0.64	0.64	0.64	0.64	0.64	0.64
Salt	0.10	0.10	0.10	0.10	0.10	0.10	0.10
Soybean oil	2.30	1.52	1.52	1.52	1.52	1.52	1.52
L-Glutamic acid, 99%	0.00	0.85	0.85	0.85	0.85	0.85	0.85
Choline chloride, 60%	0.10	0.10	0.10	0.10	0.10	0.10	0.10
Starch	0.00	0.40	0.34	0.28	0.22	0.16	0.10
L-Lysine HCl	0.34	0.32	0.32	0.32	0.32	0.32	0.32
L-Methionine	0.20	0.24	0.24	0.24	0.24	0.24	0.24
Potassium carbonate	0.00	0.28	0.28	0.28	0.28	0.28	0.28
Sodium bicarbonate	0.25	0.25	0.25	0.25	0.25	0.25	0.25
L-Glycine, 98,5%	0.00	0.12	0.12	0.12	0.12	0.12	0.12
L-Threonine, 98,5%	0.03	0.09	0.09	0.09	0.09	0.09	0.09
L-Isoleucine, 98,5%	0.03	0.11	0.11	0.11	0.11	0.11	0.11
L-Valine, 96,5%	0.00	0.08	0.08	0.08	0.08	0.08	0.08
L-Tryptophan, 98%	0.00	0.03	0.03	0.03	0.03	0.03	0.03
L-Arginine, 98,5%	0.14	0.00	0.06	0.12	0.18	0.24	0.30
Mineral Premix^1^	0.13	0.13	0.13	0.13	0.13	0.13	0.13
Vitamin Premix^2^	0.15	0.15	0.15	0.15	0.15	0.15	0.15
Salinomycin, 12%	0.06	0.06	0.06	0.06	0.06	0.06	0.06
BHT^3^	0.01	0.01	0.01	0.01	0.01	0.01	0.01
Crude Protein, %	20.77	19.04	19.04	19.04	19.04	19.04	19.04
Metabolizable energy, kcal/kg	3,200	3,200	3,200	3,200	3,200	3,200	3,200
Calcium, %	0.760	0.760	0.760	0.760	0.760	0.760	0.760
Phosphorus available, %	0.380	0.380	0.380	0.380	0.380	0.380	0.380
Sodium, %	0.203	0.203	0.203	0.203	0.203	0.203	0.203
Potassium, %	0.790	0.790	0.790	0.790	0.790	0.790	0.790
SID Arginine, %	1.203	0.940	1.000	1.060	1.120	1.180	1.240
SID Lysine, %	1.124	1.000	1.000	1.000	1.000	1.000	1.000
SID Methionine, %	0.530	0.530	0.530	0.530	0.530	0.530	0.530
SID Met. + Cys., %	0.842	0.842	0.842	0.842	0.842	0.842	0.842
SID Threonine, %	0.742	0.742	0.742	0.742	0.742	0.742	0.742
SID Tryptophan	0.202	0.202	0.202	0.202	0.202	0.202	0.202
SID Histidine, %	0.472	0.472	0.472	0.472	0.472	0.472	0.472
SID Leucine, %	1.939	1.939	1.939	1.939	1.939	1.939	1.939
SID Isoleucine, %	0.764	0.764	0.764	0.764	0.764	0.764	0.764
SID Valine, %	0.865	0.865	0.865	0.865	0.865	0.865	0.865

1 Trace mineral premix provided per kg of diet: Mn, 58.36 g; Fe, 41.68 g; Zn, 54.21 g; Cu, 8.31 g; I, 0.84 g; Se, 0.25 g.

2 Vitamin premix provided per kg of diet: vitamin A, 9,638,000 IU; vitamin D3, 2,410,000 IU; vitamin E, 36,100 IU; vitamin B1, 2.60 g; vitamin B2, 6.45 g; vitamin B6, 3.61 g; vitamin B12, 15.9 mg; vitamin K3, 1.94 g; pantothenic acid, 12.95 g; nicotinic acid, 39.20 g; folic acid, 0.90 g; biotin, 89.80 mg.

3 Antioxidant Butylhydroxytoluene.

A CP reduced diet was formulated to meet the nutritional recommendations for broilers, except for the SID Arg level, with 21.38% and 19.04% of CP, in trial 1 and 2, respectively. To reduce protein content, SID Arg and SID Lys were less supplied, in which the SID Arg:Lys ratio was decreased to 94%, in both trials (Tables 1 and 2). L-arginine (CJ Corporation, Jung-gu, Seoul, South Korea) was added to CP reduced diet at six different levels in trial 1 (0, 0.07, 0.14, 0.21, 0.28, and 0.35%) and trial 2 (0, 0.06, 0.12, 0.18, 0.24, and 0.30%) to meet the SID Arg:Lys ratios of 94, 100, 106, 112, 118, and 124%. (Tables 1 and 2).

### Broiler Performance

Broiler chickens and experimental diets were individually weighed at days 1 and 21 in trial 1 and at Days 22 and 44 in trial 2 for performance evaluation (body weight—**BW**; body weight gain—**BWG**; average daily feed intake—**ADFI**; and feed conversion ratio—**FCR**). In the case of broiler mortality, the corpse was removed, and the leftover feed was weighed to correct the ADFI.

### Skin Quality

Three birds per replicate were slaughtered by cervical dislocation at 21 days old in trial 1 and at 44 d old in trial 2. The carcass was defeathered (dry, handpicked) to remove skin samples (5 × 5 cm) from the lateral region of the left and right pelvic back, according to the methodology proposed by Bilgili et al. (1993). The skin samples were evaluated using a micrometer to measure skin thickness (**ST**) and a texturometer (Warner–Bratzler 2000, G-R Electric Company, Manhattan, KS) to measure skin strength (**SS**). The latter mechanism consisted of passing a blade over the skin, measuring the strength needed to cut off the material.

### Creatine Assays

An internal portion of the *Pectoralis profundus* (Sasami) muscle was collected from the carcass. Sasami are the slender muscles which can be found on either side of the breastbone, beneath the large breast muscle. Creatine levels in muscle (**CRM**) were assayed using an EnzyChrom Creatine Assay Kit (ECRT-100, BioAssay Systems Corporate, Hayward, CA) according to the manufacturer's protocol. In the laboratory analysis, 0.100 g of muscle samples was homogenized in 1 mL of distilled water and centrifuged at 1000 rpm for 10 minutes. The supernatant was collected and mixed with a creatine assay kit in a 96-well plate. Creatine concentrations in solution were read at OD_570nm_ using a spectrophotometer (Multiskan TM Go, Thermo Fisher Scientific Inc., Waltham, MA). The creatine standard range of the assay was 0 to 1 μM. The data were converted to ppm by a conversion factor of 0.131 according to the manufacturer's protocol.

Blood samples were collected from birds at 44 d old only in trial 2 to assess the creatine levels in serum (**CRS**). The blood samples were collected after a fasting period of 12 h by the chickens, placed in CAT Serum Activator Coagulation vacuum tubes and sent immediately to an analysis laboratory (ViçosaLab, Viçosa, Minas Gerais, Brazil) for CRS quantification, in μmol/L.

### Statistical Analysis

Data were analyzed by one-way ANOVA using ExpDes.pt from the R statistical package (R Software v.4.0.4). Dunnett's test (*P* ≤ 0.05) was used to compare each variable from treatments with diets reduced in CP to the control group. Additionally, linear and quadratic regressions were performed to model and analyze the relationships between the variables assessed and the ratios of SID Arg:Lys. When quadratic responses were detected, the optimal SID Arg:Lys ratio was calculated by taking the first derivative of the quadratic equation and subsequently estimating 95% of the maximum responses. The control treatment was not considered in the regression analysis.

## RESULTS

The SID Arg:Lys ratios in diets reduced in CP did not affect the ADFI of broiler chickens in trial 1 (*P* = 0.394) and trial 2 (*P* = 0.702) (Table 3). There were no differences between the treatments and the control group in the ADFI of broilers in either trial (*P* > 0.05) (Table 3).

**Table 3:** Means of average daily feed intake (ADFI), body weight (BW), body weight gain (BWG) and feed conversion ratio (FCR) of broilers fed diets with different SID Arg:Lys ratios and control diets in trials 1 and 2.

Traits	SID Arg: Lys ratio (%)							*P* value			SEM
	94	100	106	112	118	124	Control	ANOVA	Lin	Quad	
	Trial 1–From 01 to 21 days old										
ADFI (kg)	1.173	1.154	1.147	1.175	1.160	1.176	1.183	0.394	-	-	0.006
BW^1^ (kg)	0.944*	0.955*	0.959*	0.982	0.983	0.999	1.001	<0.001	<0.001	0.890	0.004
BGW^2^ (kg)	0.907*	0.918*	0.922*	0.945	0.946	0.961	0.963	<0.001	<0.001	0.883	0.004
FCR^3^ (kg/kg)	1.294*	1.257	1.246	1.243	1.227	1.239	1.228	0.038	0.038	0.096	0.006
	Trial 2–From 22 to 44 days old										
ADFI (kg)	3.994	4.045	4.103	4.058	4.033	4.061	4.019	0.702	-	-	0.016
BW^4^ (kg)	3.263*	3.305*	3.411	3.427	3.422	3.466	3.477	<0.001	<0.001	0.146	0.015
BGW^5^ (kg)	2.307*	2.347*	2.451	2.468	2.464	2.507	2.518	<0.001	<0.001	0.157	0.015
FCR^6^ (kg/kg)	1.732*	1.724*	1.674*	1.645	1.637	1.623	1.598	<0.001	<0.001	0.328	0.008

⁎ Means followed by an asterisk on the same line differ from the control group by Dunnett's test (*P* ≤ 0.05).

1 Y = 0.7727857 + 0.0018119X; R² = 0.96.

2 Y = 0.7354857 + 0.0018119X; R² = 0.96.

3 Y = 1.4425562 – 0.0017557X; R² = 0.72.

4 Y = 2.6661500 + 0.0065710X; R² = 0.86.

5 Y = 1.7135690 + 0.0065160X; R² = 0.87.

6 Y = 2.1047260 – 0.0039650X; R² = 0.93. Y is the response variable, and X is the SID Arg: Lys ratio. SEM = Standard Error of the Mean.

The increasing ratios of SID Arg:Lys in diets reduced in CP linearly increased the BW and BWG of birds in trials 1 and 2 (*P* < 0.001) (Table 3). Furthermore, the FCRs of broilers linearly decreased with increasing ratios of SID Arg:Lys in diets and were reduced in CP content in trial 1 (*P* = 0.038) and trial 2 (*P* < 0.001) (Table 3).

By comparing each SID Arg:Lys ratio in diets reduced in CP with the control group (Table 3), the BW and BWG of birds were lower (*P* ≤ 0.05) in the treatments with diets meeting the ratios of 94, 100, and 106% SID Arg:Lys, whereas it did not differ (*P* > 0.05) in diets meeting the ratios of 112, 118, and 124%, in trial 1. Additionally, the FCR of birds was worse than the ones from control (*P* ≤ 0.05) when were fed diets with 94% of SID Arg:Lys ratio, and it did not differ in the others SID Arg:Lys ratios (100, 106, 112, 118, and 124%) (*P* > 0.05).

In trial 2, only the diets with 94 and 100% of SID Arg:Lys ratio showed lower BW and BWG (*P* ≤ 0.05), whereas the ratios of 106, 112, 118, and 124% shower similar results than control group (*P* > 0.05) (Table 3). Furthermore, when birds were fed diets with 94, 100, and 106% of SID Arg:Lys ratio, the FCR was worse than the ones from control group (*P* ≤ 0.05), whereas the birds fed diets with 112, 118, and 124% SID Arg:Lys showed similar FCR (*P* > 0.05) (Table 3).

The increasing ratios from 94 to 124% of SID Arg:Lys linearly increased the CRM of broilers in trial 1 (*P* < 0.001) (Table 4). However, there was a quadratic effect on the CRM of broilers within the SID Arg:Lys ratios in trial 2 (*P* = 0.001), with an optimization point at the estimated level of 112% SID Arg:Lys (Table 4).

**Table 4:** Means of creatine levels in muscle (CRM) and serum (CRS) of samples of breast and blood collected from broilers at 21 and 44 d old that were fed diets with different SID Arg:Lys ratios and control diets in trials 1 and 2.

Traits	SID Arg: Lys ratio (%)							*P* value			SEM
	94	100	106	112	118	124	Control	ANOVA	Lin	Quad	
	Trial 1–21 d										
CRM^1^ (ppm)	0.151*	0.153*	0.160*	0.184*	0.234	0.265*	0.223	<0.001	<0.001	0.001	0.006
	Trial 2–44 d										
CRM^2^ (ppm)	0.090	0.096	0.099	0.110*	0.111*	0.095	0.096	<0.001	0.008	0.001	0.002
CRS^3^ (μmol/L)	92.6	97.7	100.1	124.6	138.6	135.1	116.1	<0.001	<0.001	0.959	3.390

⁎ Means followed by an asterisk on the same line differ from the control group by Dunnett's test (*P* ≤ 0.05).

1 Y = −0.2448057 + 0.0040014X; R^2^ = 0.88.

2 Y = −0.6149000 + 0.0128300X – 0.00005454 × 2; R^2^ = 0.71.

3 Y = −71.918100 + 1.7129000X; R^2^ = 0.89. Y is the response variable, and X is the SID Arg:Lys ratio. SEM = Standard Error of the Mean.

The contrast between the treatments with reduced CP and the control group showed that in trial 1, birds fed diets until the ratio of 112% SID Arg:Lys showed lower CRM than birds from the control group (*P* ≤ 0.05), whereas birds fed a diet meeting the ratio of 124% SID Arg:Lys showed higher CRM (*P* < 0.05) (Table 4). In trial 2, only the chickens fed diets meeting SID Arg:Lys ratios of 112 and 118% showed higher CRM than the chickens from the control group (*P* ≤ 0.05) (Table 4).

The CRS of broilers at 44 days old showed a linear increase as the ratio of SID Arg:Lys increased in diets reduced in CP content (*P* < 0.001) (Table 4). There were no differences in CRS between birds from the treatment and control groups (*P* > 0.05).

The ST of broilers linearly increased in trials 1 and 2 (*P* < 0.001) when the ratios of SID Arg:Lys increased from 94 to 124% in diets reduced in CP content (Table 5).

**Table 5:** Means of skin thickness (ST) and skin strength (SS) of skin samples collected from broilers at 21 and 44 d old and were fed diets with different SID Arg:Lys ratios and control diets in trials 1 and 2.

Traits	SID Arg: Lys ratio (%)							*P* value			SEM
	94	100	106	112	118	124	Control	ANOVA	Lin	Quad	
	Trial 1–21 d										
ST^1^ (mm)	0.489*	0.537*	0.622	0.688*	0.815*	0.955*	0.596	<0.001	<0.001	0.002	0.019
SS (mm)	8.269	9.111	10.478	9.820	9.088	7.928	9.320	0.108	-	-	0.264
	Trial 2–44 d										
ST^2^ (mm)	0.669*	0.861*	0.898	1.063	1.124*	1.211*	0.990	<0.001	<0.001	0.159	0.024
SS^3^ (mm)	4.545*	4.036*	5.154*	7.053*	7.888	10.171	9.963	<0.001	<0.001	0.059	0.391

⁎ Means followed by an asterisk on the same line differ from the control group by Dunnett's test (*P* ≤ 0.05).

1 Y = −0.9921810 + 0.0153786X; R^2^ = 0.97.

2 Y = −0.9313000 + 0.0174500X; R^2^ = 0.97.

3 Y = −15.111120 + 0.1980300X; R^2^ = 0.90. Y is the response variable, and X is the SID Arg:Lys ratio. SEM = Standard Error of the Mean.

By contrasting the birds from treatments with reduced CP and those from the control group, in trial 1, the ST was lower (*P* ≤ 0.05) in the SID Arg:Lys ratios of 94 and 100%, it was similar (*P* > 0.05) in the ratio of 106%, and it was higher (*P* ≤ 0.05) in the ratios of 112, 118, and 124% (Table 5). In trial 2, diets with ratios of 94 and 100% showed lower ST than control group (*P* ≤ 0.05), whereas diets with 106 and 112 showed similar ST (*P* > 0.05), and diets with 118 and 124% shower higher ST (*P* ≤ 0.05) (Table 5).

The SID Arg:Lys ratios did not influence the SS of broilers in trial 1 (*P* = 0.108) (Table 5). However, the SS linearly increased as the increasing ratios of SID Arg:Lys in diets decreased in CP in trial 2 (*P* < 0.001) (Table 5). When comparing the treatments to the control group, the SS of broilers were lower when they were fed diets meeting the ratios of 94, 100, 106, and 112% SID Arg:Lys (*P* ≤ 0.05) in trial 2 (Table 5).

## DISCUSSION

Chickens fed diets reduced in CP content would not be expected to be AA-deficient for meeting all essential AA requirements, according to Rostagno et al. (2017), except for Arg. Therefore, the effects of treatments on broiler performance should be attributed to different SID Arg:Lys ratios. Rostagno et al. (2017) recommended a ratio of 107% SID Arg:Lys in broiler diets. However, our findings demonstrated that when there was a reduction in CP of diets, broiler performance linearly improved as the ratio of SID Arg:Lys increased, with the highest ratio of 124% showing the best results for BW, BWG and FCR. Therefore, L-arginine supplementation seems to enhance chicken performance in Arg-deficient diets. Such outcomes support previous research findings that L-arginine supplementation in Arg-deficient diets leads to an increase in BW and, consequently, BWG of broiler chickens (Xu et al., 2018; Yu et al., 2018 Castro et al., 2019). However, the authors reported that Arg-excessive diets had a nonpositive effect on the growth performance of animals by decreasing BW and BWG. We did not notice a detrimental ratio of SID Arg:Lys in diets reduced in CP, which indicates that the SID Arg in diets was not overly supplied, even at the highest SID Arg:Lys ratio (124%).

There are multiple ways by which Arg acts on the growth performance of broiler chickens: it acts as a monometer in the protein chain, stimulates anabolic hormone release, and activates the TOR signaling pathway. Tsugawa et al. (2019) conducted mouse experiments to analyze the mechanism regulating arginine-induced IGF-1 secretion. Although the mechanism has not been fully elucidated, Arg seems to induce IGF-1 secretion by either inducing GH secretion and consequently stimulating IGF-1 translation and secretion or releasing IGF-1 retention in the endoplasmic reticulum. Furthermore, Yu et al. (2018) reported an increase in IGF-1 levels in the serum of broilers at 42 d old as the dietary arginine level increased. The GH/IGF axis is a key mechanism for skeletal muscle hypertrophy due to its involvement in numerous anabolic and catabolic events (Frost and Lang, 2012).

Target of rapamycin (**TOR**) signaling pathway activation consists of a phosphorylation cascade, especially the phosphorylation of TOR, ribosomal protein S6 kinase (**S6K1**), and eukaryotic initiation factor (**eIF**)-4E-binding protein-1 (**4E-BP1**), which activate the eIF4F complex, mediating the binding of mRNA to the ribosomal complex (Yonezawa et al., 2004). Yuan et al. (2015) reported that the mRNA abundance levels of TOR, 4E-BP1, and S6K1 increased as the extracellular concentrations of L-Arg increased in embryo chicken cells cultured in vitro. Similarly, Yuan et al. (2016) concluded that increasing the dietary level of L-Arg increased the liver fractional protein synthesis rate and fractional protein gain rate of laying hens by upregulating the gene expression of the TOR signaling pathway. Therefore, in our study, the growth performance results may be attributed to the role of Arg in protein synthesis contributing to protein structure, as well as via IGF-1 and TOR pathway activation.

Contrasting the treatments with diets reduced in CP with the control diet, we noticed that the BW and BWG of birds did not differ from the ratios of 112 and 106% of SID Arg:Lys, in trial 1 and 2, respectively. However, these diets with reduced CP supplied less SID Arg and SID Lys than control diet, which indicates that the control diet could have been formulated with excess of those AAs.

Arg supplementation has been shown to be effective in CR maintenance in broilers fed Arg-deficient diets, as Arg is a precursor of CR and cannot be synthesized efficiently by birds (Chamruspollert et al., 2002). Our findings showed that increasing the ratios of SID Arg:Lys in diets reduced CP and increased CRM. These results are supported by DeGroot et al. (2018), who demonstrated that an Arg-deficient basal diet containing 0.84% SID Arg was detrimental to the growth performance of broilers by producing disruptions to CR and PCR levels in muscle. However, when the authors supplied 0.16 and 0.32% L-arginine to this Arg-deficient diet, CR and PCR levels in muscle proportionally increased.

In the present study, the CRM of broilers was affected in different ways between both trials, where the increase in SID Arg:Lys ratios in diets reduced in CP linearly increased the CRM of chickens in trial 1 and quadratically increased the CRM of birds in trial 2. These results can be explained by the difference in CR requirements of broilers during their growth. The current lineages of broiler chickens are the result of successful genetic selection programs to achieve rapid growth, improvements in body conformation, and a consequent reduction in animal slaughter age (Zuidhof et al., 2014). Consequently, the nutritional requirements are higher in the starter phase than in the finisher phase. Thus, the highest ratio of 124% SID Arg:Lys in trial 1 (from 1 to 21 d old) was beneficial to the CRM of birds since the CR demand was being met. Conversely, the highest ratio of 124% SID Arg:Lys in trial 2 (from 22 to 44 d old) was detrimental to the CRM of broilers, which indicates that Arg was overly supplied, exceeding CR demands and downregulating CR synthesis. This hypothesis was confirmed by Hasegawa et al. (2017), who reported a decrease in hepatic AGAT activities as the dietary Arg levels increased from deficient to excessive. AGAT is the principal regulatory site and rate-limiting step in the biosynthesis of CR. Thus, when Arg was deficient in diets, it became the limiting AA for CR synthesis, increasing the AGAT activity to meet CR demands. Once the Arg dietary level was increased to exceed the CR requirements of broilers, AGAT activities decreased through a downregulatory mechanism.

Analyzing the contrast between the treatments with diets reduced in CP and the control group, in trial 1, we found that broilers fed diets with ratios lower than 118% of SID Arg: Lys showed lower CRM than birds from the control group, whereas the ones fed diets with the highest ratio of 124% SID Arg: Lys showed higher CRM. Interestingly, the ratio of 118% SID Arg:Lys in the diet was reduced in CP-supplemented 1.357% SID Arg, similar to the control diet, which supplied 1.344% SID Arg, whereas the ratio of 124% SID Arg:Lys in the diet was reduced in CP-supplemented 1.426% SID Arg. Thus, the increase in SID Arg levels seems to increase the CRM of broilers from 01 to 21 d old.

In trial 2, only the ratios of 112 and 118% SID Arg:Lys showed higher CRM than control group. The ratios of 112 and 118% SID Arg:Lys supplied 1.12 and 1.18% of SID Arg, respectively, whereas the control diet supplied 1.203% of SID Arg. Therefore, levels above 1.18% of SID Arg could be in excess, decreasing the CRM through a downregulatory mechanism in CR synthesis.

In the present study, the CRS of birds also increased as the SID Arg:Lys ratio increased. Such results are supported by DeGroot et al. (2018), who observed an increase in CRS as the Arg dietary levels increased from 0.84 to 1.16% of SID Arg. Such outcomes are supported by the fact that the pathway of CR synthesis occurs primarily in the liver and kidneys, which is released into the bloodstream and then taken up by muscle fibers by a CR transporter (Bender, 2012). Thus, an increase in dietary Arg may result in an increase in CR biosynthesis and, consequently, an increase in CR released into the bloodstream.

The participation of Arg in skin quality improvement has not been fully elucidated since there are many contradictory results about the role of Arg in Pro biosynthesis. Recently, studies have shown that, in contrast to mammals, the synthesis of Pro from Arg in birds is limited because of low arginase activity in their tissues. Arginase hydrolyzes Arg to ornithine (**Orn**), and the latter is converted to Pro by the enzyme ornithine-aminotransferase (**OAT**) (Wu, 2018; He et al., 2021). However, Furakawa et al. (2021) carried out a study to investigate the synthesis of polyamines from Arg and Pro in chickens and reported that mitochondrial arginase located in bird kidneys can hydrolyze Arg into Orn. These findings support previous research that indicated the presence of key enzymes responsible for Pro biosynthesis from Arg in the liver and kidneys of chickens, i.e., arginase, OAT, and pyrroline-5-carboxylic acid (**P5C**) reductase (Vecchio and Kalman, 1968; Austic and Nesheim, 1971). Additionally, Klain and Johnson (1962) demonstrated that administration of labeled arginine-C14 injections in chickens resulted in the labeling of Pro in the liver, which indicates a direct conversion of Arg to Pro. As Pro is an important structure of collagen, the possible influence between Arg dietary levels and Pro biosynthesis may explain our results of improvements in skin quality throughout the increase in SID Arg:Lys ratios. However, more studies should be conducted to elucidate the physiological mechanism involved in the effects of Arg on broiler skins.

In conclusion, the results obtained in trial 1 demonstrate that increasing ratios of SID Arg:Lys from 94 to 124% in diets reduced in protein content linearly enhances the growth performance, creatine level in muscle and skin quality of broiler chickens from 01 to 21 d old, in which the recommended ratio is 124% of SID Arg:Lys to show better results in BW, BWG, FCR, CRM, and ST. The results obtained in trial 2 demonstrate that although the creatine level in muscle has a quadratic effect within the ratios of SID Arg:Lys in diets reduced in CP, the majority of variables assessed linearly increase as the ratios of SID Arg:Lys increase from 94 to 124%, in which the recommended ratio is 124% of SID Arg:Lys to show better results in BW, BWG, FCR, CRS ST, and SS of broiler chickens from 22 to 44 d old. More studies must be carried out to better understand the mechanisms of Arg on creatine downregulation and proline biosynthesis.

## Appendix Supplementary materials

Image, application 1

Image, application 2
