# Supplementary material for: The effects of arginine supplementation through different ratios of arginine:lysine on performance, skin quality and creatine levels of broiler chickens fed diets reduced in protein content
Source: Poult Sci. 2022 Aug 27;101(11):102148. doi: 10.1016/j.psj.2022.102148 (PMC9508590; doi:10.1016/j.psj.2022.102148)
Supplement: Supplementary file 1 [file mmc1.docx]

**AUTHOR STATEMENT**

**Carlos Henrique de Oliveira:** Conceptualization, Investigation, Methodology, Writing – Original Draft, Formal analysis, Data curation. **Kelly Morais Maia Dias:** Investigation, Writing – Review & Editing. **Romário Duarte Bernardes:** Investigation, Formal analysis. **Thiago Ferreira Diana:** Investigation. **Ramalho José Barbosa Rodrigueiro:** Conceptualization, Methodology, Project administration. **Arele Arlindo Calderano:** Methodology, Writing – Review & Editing. **Luiz Fernando Teixeira Albino:** Methodology, Supervision, Project administration, Funding acquisition.
