# Supplementary material for: The effects of arginine supplementation through different ratios of arginine:lysine on performance, skin quality and creatine levels of broiler chickens fed diets reduced in protein content
Source: Poult Sci. 2022 Aug 27;101(11):102148. doi: 10.1016/j.psj.2022.102148 (PMC9508590; doi:10.1016/j.psj.2022.102148)
Supplement: Supplementary file 2 [file mmc2.pdf]

Certificate of Elsevier Language Editing Services

ELSEVIER

The following article was edited by Elsevier Language Editing Services:

"The effects of arginine supplementation through different ratios of arginine: lysine on performance, skin quality and creatine levels of broiler chickens fed diets reduced in protein content."

Authored by:

Carlos Henrique de Oliveira

Date: 28-Apr-2022

Serial number: LE-238404-4B7157FFFBC0
